# Estimating epidemiological parameters using diagnostic testing data from low pathogenicity avian influenza infected turkey houses

**DOI:** 10.1038/s41598-021-81254-z

**Published:** 2021-01-15

**Authors:** Peter J. Bonney, Sasidhar Malladi, Amos Ssematimba, Erica Spackman, Mia Kim Torchetti, Marie Culhane, Carol J. Cardona

**Affiliations:** 1 Secure Food Systems Team, Department of Veterinary and Biomedical Sciences, University of Minnesota, Saint Paul, MN USA; 2 Department of Mathematics, Faculty of Science, Gulu University, Gulu, Uganda; 3 Southeast Poultry Research Laboratory, United States National Poultry Research Center, United States Department of Agriculture, Agricultural Research Service, Athens, GA USA; 4 National Veterinary Services Laboratories, Diagnostics and Biologics, Veterinary Services, Animal and Plant Health Inspection Service, United States Department of Agriculture, Ames, IA USA

**Keywords:** Applied mathematics, Statistics, Computational biology and bioinformatics

## Abstract

Limiting spread of low pathogenicity avian influenza (LPAI) during an outbreak is critical to reduce the negative impact on poultry producers and local economies. Mathematical models of disease transmission can support outbreak control efforts by estimating relevant epidemiological parameters. In this article, diagnostic testing data from each house on a premises infected during a LPAI H5N2 outbreak in the state of Minnesota in the United States in 2018 was used to estimate the time of virus introduction and adequate contact rate, which determines the rate of disease spread. A well-defined most likely time of virus introduction, and upper and lower 95% credibility intervals were estimated for each house. The length of the 95% credibility intervals ranged from 11 to 22 with a mean of 17 days. In some houses the contact rate estimates were also well-defined; however, the estimated upper 95% credibility interval bound for the contact rate was occasionally dependent on the upper bound of the prior distribution. The estimated modes ranged from 0.5 to 6.0 with a mean of 2.8 contacts per day. These estimates can be improved with early detection, increased testing of monitored premises, and combining the results of multiple barns that possess similar production systems.

## Introduction

Outbreaks of low pathogenicity avian influenza (LPAI) can be highly disruptive to the poultry industry. LPAI can place a heavy financial burden on producers due to the reductions in egg production, decreases in bird market weight, and increases in mortality that can occur in infected flocks as well as costs stemming from intensive cleaning and extended downtime^1^. In addition, H5 and H7 LPAI strains can not only mutate into highly pathogenic avian influenza, a devastating disease in domestic poultry that can cause near 100% mortality in infected flocks^2^, but their presence in domesticated poultry can also result in significant trade restrictions. Thus, it is critical to limit the spread of virus during an outbreak.

Epidemiological investigation is an important part of any LPAI outbreak response. Transmission of LPAI between poultry premises is primarily driven by human activity, for example live bird movements, rendering truck visits to multiple premises, or equipment sharing^1^. Epidemiological investigation can identify the specific activities responsible for disease spread, allowing for effective mitigation strategies to be implemented that target a specific pathway during an outbreak. The identification of these pathways can also improve understanding of transmission routes, knowledge that can be used to inform future outbreak response. Furthermore, tracing efforts performed as part of an epidemiological investigation can highlight previously unidentified at-risk premises.

Information on the time of infection for a house or premises can assist epidemiological investigations by allowing investigators to focus on a specific timeframe, improving the precision of the pathway analysis and tracing efforts. Mathematical modeling can provide such information, estimating a range of possible virus introduction times from outbreak data^3–7^. Here, the time of virus introduction is estimated using diagnostic testing data from turkey flocks infected during a 2018 LPAI H5N2 outbreak in the state of Minnesota in the United States (U.S.). The likelihood of observing the diagnostic test results given a time of virus introduction was estimated using simulated output from a stochastic disease transmission model.

This comparison of simulated and observed data can also be used to reduce uncertainty in the internal parameters of the simulation model, a process known as inverse modelling^8^. There is substantial uncertainty in the adequate contact rate (also called the transmission parameter) for LPAI in turkeys, which determines the rate of spread in the disease transmission model. To address this uncertainty, the adequate contact rate was jointly estimated with the time of virus introduction for the LPAI infected turkey flocks in Minnesota.

To reduce uncertainty and provide disease transmission estimates applicable to epidemiological investigations, we explored an approach to estimate the time of virus introduction and adequate contact rate using diagnostic testing data from LPAI infected turkey flocks. Results are presented for the houses testing virus positive on one of the eight meat-type turkey premises infected during the 2018 LPAI H5N2 outbreak in Minnesota. The performance of the estimation approach was evaluated by applying the method to diagnostic testing data simulated with known parameters for several combinations of scenarios including high and low contact rate scenarios, scenarios varying the time of exposure relative to the sampling time of the first test, and scenarios varying by the amount and frequency of testing performed. Lastly, the method validation was extended to assess the effect of combining the likelihoods from multiple houses on the parameter estimates.

## Materials and methods

### Outbreak data

Birds on eight premises, all meat-type turkey premises, tested positive by real-time reverse transcription polymerase chain reaction (rRT-PCR) during the 2018 LPAI H5N2 outbreak in Minnesota. Diagnostic test results were available for each of the 33 barns that tested positive across the eight premises from the time of detection until the birds were sent to processing as part of controlled marketing^9^. The current article focuses on the estimated time of virus introduction and adequate contact rate results for a single premises, the 2nd premises detected in Kandiyohi county in Minnesota (Kandiyohi 2). Results from the other premises have been reported^10^. All five barns on Kandiyohi 2 tested positive for virus. The data consisted of the number of birds in each barn, the date swabs for rRT-PCR or serum samples were taken, and the number of positive diagnostic test results. Swabs for rRT-PCR were taken from live birds and tested in pools of 10 or 11 swabs. Serology testing consisted of 10 serum samples tested individually by either agar gel immunodiffusion (AGID) or enzyme-linked immunosorbent assay (ELISA). The Kandiyohi 2 testing data is provided in Supplementary Table S1. All samples were collected and tested under the guidance and authority of the Code of Federal Regulations (CFR) enacted to guide avian influenza surveillance in poultry as outlined in 9 CFR parts 145, 146, 147 and 56^11^.

### Stochastic within-house LPAI transmission model

The infection prevalence of LPAI and seroprevalence over time simulated from the stochastic within-house transmission model were used to estimate the likelihood of observing the diagnostic test results given different virus exposure times and adequate contact rates. The transmission model for LPAI in broiler breeders from Bonney et al. was reparametrized for LPAI in turkeys^12^. A summary of the input parameters is given in Table 1. The transmission model tracks the number of birds in the susceptible, latently infected, infectious, recovered, removed (dead), and seroconverted states at each simulation time step, which in this case was set to 0.01 days.

**Table 1:** Input parameters for the stochastic within-house LPAI transmission model.

Parameter name/notation	Description	Distribution/value	References
Latent period distribution	Length in days of the latent period	~ Gamma (shape = 2.58, scale = 0.24); mean = 0.63 days, standard deviation = 0.39 days	^15–17^
Infectious period distribution	Length in days of the infectious period	~ Gamma (shape = 4.04, scale = 2.92); mean = 11.78 days, standard deviation = 5.86 days	^3,15–19^
Time to seroconversion distribution	Length in days of the time from infection to seroconversion	~ Gamma (shape = 3.56, scale = 1.63); mean = 5.80 days, standard deviation = 3.07 days	^20–23^
$P_{mort}$	Proportion of birds that die following exposure to LPAI	0.01	Field data
$P_{sero}$	Proportion of birds that seroconvert following exposure to LPAI	0.99	^19^

The number of initially infected birds in a house was dependent on whether the house was the first infected on the premises, as deduced from diagnostic testing data. In the house that first tested positive for virus, each transmission run began with a single latently infected bird. The number of initially infected birds was increased for the houses that tested positive for virus at a later date based on the assumption that more virus would be present on the premises and thus likely transmitted to more susceptible birds in neighboring houses. For these houses, the number of initially infected birds was randomly sampled from the integers between one and ten. The number of birds initially infected in non-index barns during an outbreak is unknown given potential on-farm spread due to movement of people or equipment and could be higher than the baseline assumption of one to ten^1^. Due to the uncertainty in this parameter a sensitivity scenario was evaluated where the interval for the number of initially infected birds was increased from one to fifty. For the results and discussion of this sensitivity analysis see Supplementary Table S9 and Supplementary Table S10.

In each proceeding time step, the number of infected birds was simulated from a binomial distribution as follows. Let β be the adequate contact rate, $N_{I} \left( t \right)$ be the number of infectious birds at time step $t$, and $N\left( t \right)$ be the number of birds alive at time step $t$. The probability of infection in the time interval $\left( {t, t + \Delta t} \right)$, $p_{inf} \left( {t,\Delta t} \right)$, is given by the equation^13,14^:

$$p_{inf} \left( {t,\Delta t} \right) = 1 - e^{{\left( { - \beta \frac{{N_{I} \left( t \right)}}{N\left( t \right)}\Delta t} \right)}}$$

The length of the time step $\Delta t$ was set to 0.01 days in this analysis. Next, let $N_{S} \left( t \right)$ be the number of susceptible birds at time step $t$. The number of newly infected birds in the time interval $\left( {t, t + \Delta t} \right)$, $N_{new\_cases} \left( {t, \Delta t} \right)$, is modeled by the binomial distribution^13^:

$$N_{new\_cases} \left( {t,\Delta t} \right) \sim Binomial\left( {N_{S} \left( t \right), p_{inf} \left( {t,\Delta t} \right)} \right)$$

As constructed, any increase in the contact rate increases the probability that a susceptible bird is infected in the simulation time step, resulting in a faster rate of disease spread.

Random latent period and infectious period lengths were generated for each infected bird from distributions modeling their respective lengths. Birds transitioned out of the latent and infectious states in the first simulation time step where the randomly generated length was exceeded. The parameters of the latent and infectious period length distributions, assumed to be gamma distributed, were estimated from inoculation study data using a maximum likelihood estimation method. Data involving both H5 and H7 LPAI strains were included due to insufficient data on turkeys inoculated with H5 LPAI strains. The inoculation studies used in the estimation of the latent period distribution include Pillai et al., Iqbal et al., and Pantin-Jackwood et al.^15–17^ and those for the infectious period distribution include Pillai et al., Saenz et al., Iqbal et al., Comin et al., Pantin-Jackwood et al., and Spackman et al.^3,15–19^.

The proportion of birds that die from infection was set to 0.01 based on the low mortality observed in houses infected during this Minnesota LPAI outbreak. The birds designated to die from infection transition from the infectious to the removed state, while the other birds transition to the recovered state. Once a bird is in the removed or recovered state, it remains there for the rest of the simulation.

The transition into the seroconverted state occurred as follows. Once a bird was infected, a random number was generated from a distribution modeling the time to seroconversion. A bird transitioned into the seroconverted state in the first time step where this length was exceeded. The time to seroconversion was assumed to be gamma distributed. The parameters of this distribution were estimated from inoculation studies using a maximum likelihood method. Data involving any LPAI virus strain were included due to a scarcity of viable data. The studies used were Dundon et al., Morales, Homme et al., and Preskenis, which involved H4, H6, H7, and H9 LPAI strains^20–23^. The proportion of birds that seroconvert was set to 0.99 based on the results of Spackman et al. (2010) in which 88/89 (99%) of the turkeys inoculated with twelve North American H7 LPAI isolates had detectable antibody by day 18 to 21 post-inoculation^19^.

Due to high uncertainty in the parameter estimate for the proportion of birds that die due to infection, a sensitivity analysis was performed where the proportion of birds dying due to disease was set to 0 and 0.04. The Kandiyohi 2 results were identical to the baseline results in the sensitivity analysis, suggesting the estimates are robust to changes in the proportion of infected birds that die due to disease. This is to be expected since only live birds were sampled for testing. There is uncertainty in other parameter estimates such as the shape and scale of the time to seroconversion distribution. More work is required to characterize this uncertainty in the form of prior distributions for these parameters. However, this was reserved for future work in order to focus the paper on the estimation method for the time of virus introduction and adequate contact rate.

### Estimation of the time of virus introduction and adequate contact rate

The time of virus introduction and the adequate contact rate were estimated for each poultry house using a Bayesian approach. The likelihood of observing the diagnostic test results was estimated given output simulated from the stochastic within-house LPAI transmission model for different virus introduction time and contact rate pairs. Similar to Pinsent et al. (2014), the likelihood of observing a test result was modeled with the binomial distribution^4^. A summary of the notation used in the following derivation of the estimation method is given in Supplementary Table S2.

The probability of observing a pooled rRT-PCR test result, with all swabs taken from live birds, was determined as follows. First, let $N_{pcr,j}^{swabs}$ be the number of swabs per pool in rRT-PCR test event *j*. A test event was defined as the testing performed on samples taken at the same time. In the case of Kandiyohi 2, the rRT-PCR test events were comprised of either 3 pooled samples of 11 swabs each or 1 pooled sample of 11 swabs. Let $\rho_{I,ij} \left( {\beta , t_{intro} } \right)$ be the infection prevalence at the time of sampling for test event *j*. This prevalence was estimated from the *i*th LPAI transmission model run simulated with a given contact rate $\beta$ and time of virus introduction $t_{intro}$. The probability of including at least one swab from an infectious bird in a single pooled sample taken as part of test event *j* and based on simulation run *i*, a probability notated as $p_{pcr,ij}$, is

$$p_{pcr,ij} = 1 - \left( {1 - \rho_{I,ij} \left( {\beta , t_{intro} } \right)} \right)^{{N_{pcr,j}^{swabs} }}$$

Next, let $N_{pcr,j}^{tests}$ be the total number of pooled rRT-PCR samples tested as part of test event *j* and $N_{pcr,j}^{pos}$ be the number of pooled samples that tested positive in test event *j*. Both $N_{pcr,j}^{tests}$ and $N_{pcr,j}^{pos}$ were observed from the field data. Finally, let $Se_{pcr}$ be the rRT-PCR test sensitivity, in this case equal to 0.90^24^. The probability of observing $N_{pcr,j}^{pos}$ given contact rate $\beta$ and time of virus introduction $t_{intro}$ and based on output from transmission run *i* is

$$P_{i} \left( {N_{pcr,j}^{pos} | \beta , t_{intro} } \right) = \left( {\begin{array}{*{20}c} {N_{pcr,j}^{tests} } \\ {N_{pcr,j}^{pos} } \\ \end{array} } \right)\left( {Se_{pcr} p_{pcr,ij} } \right)^{{N_{pcr,j}^{pos} }} \left( {1 - Se_{pcr} p_{pcr,ij} } \right)^{{N_{pcr,j}^{tests} - N_{pcr,j}^{pos} }}$$

The probability of observing a serology test result was determined similarly. Let $N_{sero,k}^{tests}$ be the number of serum samples taken as part of serology test event *k* and $N_{sero,k}^{pos}$ be the number of those samples that test positive, where both $N_{sero,k}^{tests}$ and $N_{sero,k}^{pos}$ were observed in the field data. Next, let $\rho_{S,ik} \left( {\beta , t_{intro} } \right)$ be the seroprevalence in the flock at the time of sampling for test event *k*, estimated from the *i*th disease transmission model simulation run given contact rate $\beta$ and time of virus introduction $t_{intro}$. Last of all, let $Se_{sero}$ be the AGID or ELISA test sensitivity, in this case set equal to 1.00^25^. A sensitivity analysis was performed for the $Se_{sero}$ parameter where the time of virus introduction and adequate contact rate were estimated from the Kandiyohi 2 diagnostic test data considering an AGID or ELISA test sensitivity of 0.98. Little difference was observed in the results of the sensitivity analysis compared to the baseline. Results are given in Supplementary Table S7 and Supplementary Table S8. The probability of observing $N_{sero,k}^{pos}$ given contact rate $\beta$ and time of virus introduction $t_{intro}$ and based on the output from the *i*th iteration of the disease transmission model is

$$P_{i} \left( {N_{sero,k}^{pos} | \beta , t_{intro} } \right) = \left( {\begin{array}{*{20}c} {N_{sero,k}^{tests} } \\ {N_{sero,k}^{pos} } \\ \end{array} } \right)\left( {Se_{sero} \rho_{S,ik} \left( {\beta , t_{intro} } \right)} \right)^{{N_{sero,k}^{pos} }} \left( {1 - Se_{sero} \rho_{S,ik} \left( {\beta , t_{intro} } \right)} \right)^{{N_{sero,k}^{tests} - N_{sero,k}^{pos} }}$$

The samples taken for rRT-PCR and serology testing were performed without replacement, meaning a hypergeometric distribution would provide a more exact probability of observing the test result. However, due to the large population size in the houses (over 7200 turkeys in each barn) compared to the size of the samples taken for testing (no more than 33 turkeys sampled for rRT-PCR and 10 turkeys sampled for serology testing), a binomial distribution can serve as an appropriate substitute. As its implementation was more straightforward, a binomial approximation was used in this analysis.

The likelihood of observing the set of diagnostic test results in a house for a given contact rate and time of virus introduction was estimated by taking the average across the 10,000 simulation iterations of the product of the individual test probabilities. Let $N_{iterations}$ be the number of transmission model simulation iterations run for each contact rate and time of virus introduction pair. Let $N_{pcr}$ be the total number of rRT-PCR test events and $N_{sero}$ be the total number of serology test events. The likelihood of observing the diagnostic test results given contact rate $\beta$ and time of virus introduction $t_{intro}$ is

$$l\left( { x | \beta , t_{intro} } \right) = \frac{{\mathop \sum \nolimits_{i = 1}^{{N_{iterations} }} \mathop \prod \nolimits_{j = 1}^{{N_{pcr} }} P_{i} \left( {N_{pcr,j}^{pos} | \beta , t_{intro} } \right)\mathop \prod \nolimits_{k = 1}^{{N_{sero} }} P_{i} \left( {N_{sero,k}^{pos} | \beta , t_{intro} } \right)}}{{N_{iterations} }}$$

The prior distributions for the contact rate and time of virus introduction were assumed to be uniform distributed. The distribution for the contact rate had a minimum of 0.1 and maximum of 6.0 contacts per day, which was set based on LPAI and HPAI contact rate estimates for turkeys in the literature^3,18,26–29^. The latest time of virus introduction that was evaluated was the time of sampling for the first positive diagnostic test result observed in the data. The earliest time of virus introduction that was evaluated was 80 days prior to the sampling time of the last diagnostic test taken from the house.

Due to the low dimensionality of this problem (two parameters to be estimated), the parameter space defined by the prior distributions was explored using a grid-based method. The contact rate was incremented by 0.1 steps while the time of virus introduction was incremented by 0.25 day steps. The likelihood was estimated for each combination of contact rate and virus introduction and then normalized to obtain a posterior distribution. Let $\pi \left( {\beta , t_{intro} } \right)$ be the prior distribution for the contact rate and time of virus introduction. The posterior distribution, $p\left( {\beta , t_{intro} |x} \right)$, was determined by

$$p\left( {\beta , t_{intro} |x} \right) = \frac{{l(x|\beta , t_{intro} )\pi \left( {\beta , t_{intro} } \right)}}{{\mathop \sum \nolimits_{\beta } \mathop \sum \nolimits_{{t_{intro} }} l\left( {x|\beta , t_{intro} } \right)\pi \left( {\beta , t_{intro} } \right)}}$$

A posterior distribution was estimated for each house following this approach.

### Multiple house estimation approach

The single house likelihoods can be conditioned on the results from other houses with similar production characteristics (e.g. similar housing structures and bird ages) with the goal of improving the parameter estimates. Houses with similar production characteristics were assumed to have the same contact rate. Therefore, the likelihoods from these other houses represent a source of additional information.

The contact rate and time of virus introduction were estimated for a given house conditioned on the contact rate likelihoods observed in houses with similar production characteristics using the following approach. A summary of the notation used for the multiple house estimation method is given in Supplementary Table S3. First, a multi-house contact rate likelihood was derived by multiplying the marginal contact rate likelihoods, estimated by summing up the likelihood across each candidate time of exposure for a given contact rate, from each house. Let $l_{h} \left( {x|\beta , t_{intro} } \right)$ be the likelihood of observing the diagnostic testing data from house *h*. Let $t_{1}$ be the earliest and $t_{max}$ be the latest time of virus introduction evaluated for house *h*. The marginal likelihood for the contact rate, $l_{h} \left( {x|\beta } \right)$, was determined by

$$l_{h} \left( {x|\beta } \right) = \mathop \sum \limits_{{t_{intro} = t_{1} }}^{{t_{max} }} l_{h} \left( {x|\beta , t_{intro} } \right)$$

Without loss of generality, suppose we are interested in estimating the time of virus introduction and contact rate from house 1, notated $h_{1}$. Suppose that the set $H$ of houses, $h_{2} , h_{3} , \ldots , h_{n}$, have similar production systems to house $h_{1}$. The multiple house contact rate likelihood, $l_{H} \left( {x|\beta } \right)$, was determined by

$$l_{H} \left( {x|\beta } \right) = \mathop \prod \limits_{{h = h_{2} }}^{{h_{n} }} l_{h} \left( {x|\beta } \right)$$

The house 1 likelihood was combined with the multi-house contact rate likelihood through multiplication. Let $l_{{h_{1} }}^{H} \left( {x|\beta , t_{intro} } \right)$ be the house 1 posterior likelihood conditioned on the contact rates from the houses in set *H*. This likelihood was determined by

$$l_{{h_{1} }}^{H} \left( {x|\beta , t_{intro} } \right) = l_{{h_{1} }} \left( {x|\beta , t_{intro} } \right)l_{H} \left( {x|\beta } \right)$$

Estimates for the contact rate and time of virus introduction were then estimated from the posterior $p_{{h_{1} }}^{H} \left( {\beta , t_{intro} |x} \right)$, where

$$p_{{h_{1} }}^{H} \left( {\beta , t_{intro} |x} \right) = \frac{{l_{{h_{1} }}^{H} \left( {x|\beta , t_{intro} } \right)\pi \left( {\beta , t_{intro} } \right)}}{{\mathop \sum \nolimits_{\beta } \mathop \sum \nolimits_{{t_{intro} }} l_{{h_{1} }}^{H} \left( {x|\beta , t_{intro} } \right)\pi \left( {\beta , t_{intro} } \right)}}$$

**Figure 1:**
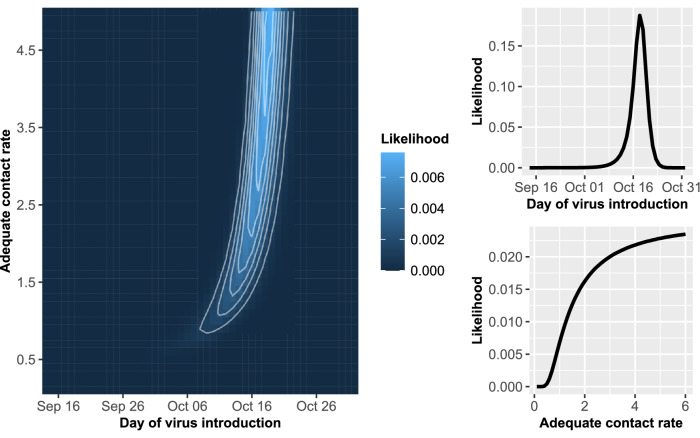
A contour plot of the posterior likelihood and plots of the marginal posterior likelihoods for the adequate contact rate and day of virus introduction estimated from the Kandiyohi 1 house 1 data.

**Figure 2:**
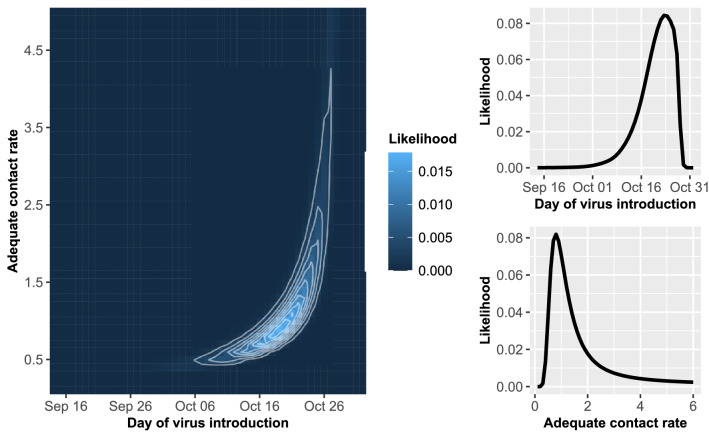
A contour plot of the posterior likelihood and plots of the marginal posterior likelihoods for the adequate contact rate and day of virus introduction estimated from the Kandiyohi 1 house 2 data.

The models were coded in R statistical software and C programming language and the analysis was performed using supercomputing resources of the Minnesota Supercomputing Institute^30,31^. Figures 1 and  2 were produced using the ‘ggplot2′ package in R^30,32^.

### Method validation

The time of virus introduction and adequate contact rate were estimated from simulated data generated with known parameters in order to validate the estimation approach. Test results were simulated for two test schedule and two contact rate scenarios for six barns, each with a different time of exposure relative to the first test. The first test schedule scenario was a replicate of the Kandiyohi 2 test schedule. The second test schedule scenario consisted of an intensive testing schedule of three pooled rRT-PCR samples of 11 swabs each and 10 serum samples tested by AGID or ELISA taken on the same day every three days. The two contact rate scenarios consisted of a slow contact rate scenario, with the contact rate set to 1.0 contacts per bird per day, and a fast contact rate scenario, with the contact rate set to 4.5 contacts per bird per day. The times of virus introduction relative to the first test day for the six barns were 19 days prior, 6 days prior, 3 days prior, 1 day after, 2 days after, and 9 days after, respectively.

The validation datasets were generated by first simulating the number of positive birds in a sample from a binomial distribution with the probability of sampling a positive bird equal to the infection prevalence or seroprevalence estimated from the LPAI transmission model. If a positive bird was sampled, the test result was simulated from a Bernoulli distribution with the probability of the sample testing positive equal to the test sensitivity. The validation datasets were formed by taking the average test results from 10,000 simulation iterations for each barn under each test schedule and contact rate scenario. The time of virus introduction and adequate contact rate were estimated from these datasets for both the single and multiple house approach.

## Results

### Estimated time of virus introduction and adequate contact rate from Kandiyohi 2 diagnostic testing data

The mode, median, and 95% credibility interval (CI) estimated from diagnostic testing data from each of the five houses on Kandiyohi 2 are given in Table 2. Figures 1 and 2 display contour plots of the posterior likelihood and marginal posterior likelihood plots for the time of virus introduction and adequate contact rate for Kandiyohi 2 house 1 and Kandiyohi 2 house 2, respectively. The marginal posterior likelihood for the time of virus introduction was parabolic in shape for each house, meaning an informative mode and 95% CI could be estimated. On average, the 95% CI’s spanned 17 days.

**Table 2:** Mode, median, and 95% CI for the date of virus introduction and adequate contact rate estimated from each house on Kandiyohi 2.

House	Estimated date of virus introduction mode (median; 95% CI)	Estimated adequate contact rate mode (median; 95% CI)
House 1	October 18 (Oct 17; Oct 10–Oct 21)	6.0 (3.9; 1.2–5.8)
House 2	October 23 (Oct 21; Oct 7–Oct 27)	0.8 (1.2; 0.5–5.0)
House 3	October 21 (Oct 20; Oct 6–Oct 26)	0.8 (1.0; 0.5–4.1)
House 4	October 22 (Oct 20; Oct 6–Oct 28)	0.5 (0.6; 0.4–1.0)
House 5	October 18 (Oct 17; Oct 11–Oct 22)	6.0 (3.9; 1.2–5.8)

An informative mode and 95% CI could not always be estimated for the adequate contact rate. In two of the houses on Kandiyohi 1, house 1 and house 2, the likelihood increased as the contact rate increased, as can be seen for house 1 in Fig. 1. For these two houses, only the lower CI bound is informative, with the upper CI bound entirely dependent on the upper bound of the prior distribution. In house 2, house 3, and house 4, on the other hand, the marginal posterior likelihood for the contact rate was parabolic in shape, allowing for more informative estimates.

### Method validation results

The mode, median, and 95% CI estimated from simulated diagnostic testing data based on the Kandiyohi 2 testing schedule for six houses with different dates of virus introduction and same adequate contact rate of 1.0 contact per day are given in Table 3. Estimates are given for the single and multiple house approach. The multiple house approach results given in the table consider the addition of one house and five houses. The results for the addition of one house are given as the average mode, median, and 95% CI estimated from all the possible house pairings. Validation results estimated from the three scenarios not represented in Table 3. ie. the results from simulated Kandiyohi 2 test data given a contact rate of 4.5 and simulated increased testing data given a contact rate of 1.0 contact per day and 4.5 contacts per day, are provided in the Supplementary Table S4, Supplementary Table S5, and Supplementary Table S6, respectively.

**Table 3:** Validation results given barns were infected 19, 6, and 3 days prior to and 1, 2, and 9 days after the date of the first test, assumed to have been performed on October 20.

Given time of virus introduction and adequate contact rate	Single barn estimates mode (median; 95% CI)	Average mode (median; 95% CI) estimated with the contact rate distribution from 1 additional house	Mode (median; 95% CI) estimated with the contact rate distribution from 5 additional barns
Oct 1;	Oct 8 (Oct 7; Sep 20–Oct 12);	Oct 7 (Oct 6; Sep 22–Oct 12);	Oct 3 (Oct 2; Sep 24–Oct 9);
1.0	0.6 (3.2; 0.6–5.8)	2.0 (2.5; 0.7–5.5)	1.1 (1.4; 0.8–3.3)
Oct 14;	Oct 21 (Oct 20; Oct 10–Oct 25);	Oct 19 (Oct 19; Oct 9–Oct 24);	Oct 16 (Oct 16; Oct 9–Oct 22);
1.0	6.0 (3.5; 0.9–5.8)	2.5 (2.8; 0.8–5.6)	1.2 (1.5; 0.8–3.5)
Oct 17;	Oct 25 (Oct 23; Oct 9–Oct 27);	Oct 24 (Oct 22; Oct 10–Oct 26);	Oct 20 (Oct 19; Oct 11–Oct 24);
1.0	1.1 (3.1; 0.7–5.8)	1.3 (2.5; 0.8–5.5)	1.1 (1.4; 0.8–3.3)
Oct 21;	Oct 27 (Oct 25; Oct 13–Oct 29);	Oct 26 (Oct 24; Oct 15–Oct 29);	Oct 23 (Oct 23; Oct 17–Oct 27);
1.0	1.1 (1.8; 0.6–5.4)	1.1 (1.7; 0.7–5.0)	1.1 (1.3; 0.8–2.6)
Oct 22;	Oct 26 (Oct 24; Oct 13–Oct 29);	Oct 26 (Oct 24; Oct 15–Oct 29);	Oct 24 (Oct 23; Oct 17–Oct 27);
1.0	1.0 (1.6; 0.6–5.0)	1.0 (1.5; 0.7–4.7)	1.1 (1.3; 0.8–2.4)
Oct 29;	Nov 5 (Nov 4; Oct 22–Nov 7);	Nov 4 (Nov 3; Oct 24–Nov 7);	Nov 1 (Oct 31; Oct 24–Nov 5);
1.0	1.3 (3.1; 0.7–5.8)	1.3 (2.5; 0.8–5.5)	1.1 (1.4; 0.8–3.3)

The time of virus introduction and adequate contact rate were estimated from simulated test data based on the Kandiyohi 2 test data and a contact rate of 1.0 contacts per day. Results are given for the single barn approach and multiple house approach.

The true estimates for the time of virus introduction and contact rate were contained by the 95% CI for each validation run under the single house estimation approach. Including the contact rate information from other houses in the multiple house approach generally both improved the accuracy and reduced uncertainty in the estimates. Similar issues with estimating an upper bound for the contact rate as encountered in the Kandiyohi 2 field data were present in the validation results. This can be observed in the results under the single barn approach in rows 1, 2, 3, and 6 in Table 3, where the upper bound for the contact rate is dependent on the bound of the prior distribution.

As expected, more accurate estimates with narrower 95% CI bounds were observed in the results of the validation scenarios with increased testing. The estimation approach also performed better when the house was exposed to virus relatively close to or after the time of the first test as compared to houses exposed multiple days prior. While the estimates for the day of virus introduction were more accurate in the higher contact rate scenarios, performance did not necessarily improve for the contact rate estimates. An informative lower 95% CI bound could be estimated for the higher contact rate scenarios, but the upper bound, and oftentimes the mode, were dependent on the upper bound of the prior distribution.

## Discussion

Monitoring flocks with diagnostic testing is an intrinsic part of outbreak management strategies like controlled marketing in LPAI infected poultry flocks. Pinsent et al. (2014) developed a method to estimate the time of virus introduction and basic reproduction number from diagnostic testing data using a binomial likelihood and output from a deterministic disease transmission model^4^. Here, diagnostic testing data from five barns infected on a premises during the 2018 LPAI H5N2 outbreak in the U.S. state of Minnesota were used to estimate the time of virus introduction and adequate contact rate for each barn. The approach used built on the Pinsent et al. (2014) method in the following ways. First, output from a stochastic disease transmission model was used to estimate the likelihood as opposed to a deterministic model. Furthermore, pooled testing in addition to individual bird testing was incorporated into the method, and the method was applied to field data as opposed to simulated data only. Most notably, the method was extended to allow for parameter estimation from the combined likelihoods of multiple houses with the goal of reducing uncertainty in the estimates.

The parameter estimates for the time of virus introduction and adequate contact rate can be used in a number of applications related to preventing widespread transmission of LPAI during an outbreak. The time of virus introduction estimates provide information on how the outbreak unfolded, which can help stakeholders evaluate the management of the outbreak and inform the response to future outbreaks. For example, the time between the estimated virus introduction time and date of detection could be used to assess the effectiveness of the active surveillance program used to monitor premises status. Most importantly, the estimates for the time of virus introduction can support epidemiologic investigations by helping stakeholders focus their efforts on a specific time window.

The adequate contact rate can be an important input parameter in epidemiological models, including the stochastic disease transmission model used in this study in which the contact rate determines the within-flock rate of disease spread. The transmission model has also been used previously to inform surveillance design and risk assessments^12,33,34^. The adequate contact rate can vary according to factors like the virus strain, bird species and age, or housing type. The rate of disease spread can have a direct impact on the effectiveness of outbreak control measures. Diseases with slow rates of spread, for example, can take longer to be detected than ones with faster rates under the same active surveillance protocol^34^. Additionally, contact rate estimates can generate insight into the risk factors for fast and slow spread, which can be used to inform intervention strategies for specific houses and premises. During this LPAI outbreak in Minnesota, some industry veterinarians requested predictions for the time until no more infectious birds would be present in infected houses based on the latest diagnostic test results. These predictions can support logistics planning, namely when to schedule the houses to be sent to processing. Moreover, the accuracy of the predictions can be improved with contact rates estimated directly from the outbreak in progress.

The only estimates for the contact rate for LPAI in turkeys identified in a literature review were from Comin et al. (2011) and Saenz et al. (2012)^3,18^. Furthermore, only in Comin et al. (2011) was the transmission parameter estimated from field data, where the spread dynamics can differ from those of the laboratory in transmission experiments^3^. As a result, there is substantial uncertainty in the turkey LPAI contact rate, which increases uncertainty in the appropriate contact rate value or distribution to use in proactive risk assessment, where it can be advantageous to model outbreak conditions with greater generality. Thus, the contact rates estimated from the turkeys flocks infected with LPAI in the field in Minnesota provide critical information on this parameter.

The estimation approach performed well for estimating the time of virus introduction, as an informative mode, upper 95% CI bound, and lower 95% CI bound could be estimated for each house on Kandiyohi 2 as well as for each of the validation simulation runs. While informative estimates could be derived for the contact rate for many houses and validation runs, for others the estimates for the mode and upper 95% CI bound were uninformative due to being dependent on the upper bound of the prior distribution. An example of the latter can be observed in the plot of the marginal posterior likelihood for the contact rate estimated from the Kandiyohi 1 house 1 diagnostic testing data given in Fig. 1. The shape of the marginal posterior is suggestive of practical non-identifiability, which refers to the inability to derive informative estimates from the model due to limited or uncertainty in the data^35^.

The validation results suggest a few strategies that can improve contact rate identifiability. First of all, greater uncertainty was observed in the validation results when the flock was close to 100% seroconverted by the time of the first test. When the flock is close to 100% seroconversion, there is little change in the test results; they quickly fall into a pattern of all negative rRT-PCR results and all seropositive serum samples. This introduces uncertainty into the contact rate estimates in that the pattern of change observed in the test results provides information on the rate of spread. For example, suppose 10 serum samples were collected five days apart. A result of zero seropositive samples followed by 10 seropositive samples would suggest faster disease spread than a result of zero seropositive samples followed by 3 seropositive samples. An example of greater uncertainty in the contact rate due to testing starting close to or after the flock is 100% seroconverted can be observed in the estimates for the validation house assumed to be exposed 19 days prior to the first test given in the first row of Table 3. All this suggests that early detection of LPAI virus in a house is important for obtaining identifiable contact rate estimates.

A second strategy that can improve contact rate estimation is to increase the amount of testing done during the outbreak. An example of improved contact rate estimates due to increased testing can be observed in the validation results for the house assumed to be exposed six days prior to the first day of testing given in Table 3 compared to the results given in Supplementary Table S4. The third strategy is to use the multiple house estimation method, which improved the contact rate estimates for the validation houses in rows 1, 2, 3, and 6 in Table 3, for example. While this strategy is far easier and less costly to implement than increasing active surveillance for early detection or increasing the amount of testing performed while monitoring flock status, it may not always be appropriate due to the houses having differing production characteristics. Detecting virus in the house well before 100% seroconversion, increasing the amount of testing performed for monitoring infected flocks, and using the multiple house estimation method all improve the quality of information on the dynamics of virus spread within the infected houses. This not only can refine the estimates for the contact rate, but also can reduce uncertainty and improve the accuracy of estimates for the time of virus introduction.

The objective in this study of estimating parameters from diagnostic testing data has the benefit of a defined likelihood function that can link output from the stochastic simulation to the observed data, i.e. the binomial likelihood function. The time of virus introduction and adequate contact rate could alternatively be estimated using a likelihood-free approach such as Approximate Bayesian Computation (ABC)^36^, a method growing in popularity and used by Guinat et al. (2018), for example, to estimate the time of virus introduction in pig herds infected with African swine fever^7^.

An advantage of the ABC approach as compared to the likelihood approach used in this analysis is that the parameter space can be sampled using Markov Chain Monte Carlo (MCMC), allowing for more efficient exploration of the parameter space. This advantage would be even more pronounced as the number of parameters to be estimated increases, since the number of unique parameter combinations would increase exponentially in the grid-based method used in the likelihood approach. However, convergence of the MCMC chains can be difficult to achieve. The main advantage of the likelihood parameter estimation approach is the ability to easily combine the likelihoods from multiple houses and estimate a combined contact rate distribution that can then be used to estimate the time of virus introduction for a given house. It was demonstrated in the validation results that the multiple house estimation method can reduce uncertainty in and improve the accuracy of the contact rate and time of virus introduction estimates. Joint estimation of the contact rate for multiple houses would be challenging under the ABC approach in that the simulated output would have to match or be close to the observed test results for multiple houses, leading to low acceptance rates. Both approaches are computationally intensive and their implementation is only feasible due to advances in and wider availability of supercomputing resources.

Estimating parameters from diagnostic testing data has utility for LPAI in poultry due to established tests that can detect virus and antibodies with high sensitivity. The estimation approach detailed in this article may be less applicable to those viruses whose diagnostic tests have low sensitivity as this would introduce greater uncertainty into the observed test results and estimated parameters. Since LPAI virus infections are often associated with low mortality, diagnostic testing is an especially important indicator for the progression of the virus through meat turkey and broiler flocks. Egg laying flocks can have an additional indicator in the form of decreased egg production. Egg production data could be combined with diagnostic testing data using an approach such as ABC to estimate the time of virus introduction or contact rate. For viruses such as highly pathogenic avian influenza or African swine fever that cause very high mortality in the host population, an approach that relies on the mortality data has the greatest utility. Examples of approaches for high mortality include Bos et al. and Guinat et al.^5,7^.

Overall, our findings demonstrate a valid approach for estimating disease transmission parameters that has been used to support epidemiological investigations of LPAI outbreaks in Missouri, North and South Carolina, and the outbreak in Minnesota^10,37^. This approach may also be deployable in response to outbreaks caused by high consequence viruses in other species and geographical locations.

## Supplementary information

Supplementary Information.

## Data Availability

The diagnostic testing data analyzed in this study are available in Supplementary Table S1 in the Supplementary Materials.

## References

[1] Halvorson, D. A. In *Avian Influenza* (ed David E. Swayne) Ch. 23, 513–536 (Blackwell Publishing Ltd., 2008).

[2] Halvorson DA (2002). The control of H5 or H7 mildly pathogenic avian influenza: a role for inactivated vaccine. Avian Pathol..

[3] Comin A, Klinkenberg D, Marangon S, Toffan A, Stegeman A (2011). Transmission dynamics of low pathogenicity avian influenza infections in Turkey flocks. PLoS ONE.

[4] Pinsent A, Blake IM, White MT, Riley S (2014). Surveillance of low pathogenic novel H7N9 avian influenza in commercial poultry barns: detection of outbreaks and estimation of virus introduction time. BMC Infect. Dis..

[5] Bos ME (2007). Estimating the day of highly pathogenic avian influenza (H7N7) virus introduction into a poultry flock based on mortality data. Vet. Res..

[6] Stegeman A, Elbers AR, Bouma A, de Smit H, de Jong MC (1999). Transmission of classical swine fever virus within herds during the 1997–1998 epidemic in The Netherlands. Prevent. Vet. Med..

[7] Guinat C (2018). Inferring within-herd transmission parameters for African swine fever virus using mortality data from outbreaks in the Russian Federation. Transbound. Emerg. Dis..

[8] Hartig F, Calabrese JM, Reineking B, Wiegand T, Huth A (2011). Statistical inference for stochastic simulation models–theory and application. Ecol. Lett..

[9] U.S. Department of Agriculture, A. P. H. I. S. *Code of Federal Regulations* (ed Code of Federal Regulations) (U.S. Government Publishing Office, 2019).

[10] USDA-APHIS. Epidemiologic and other analyses of avian influenza infected poultry flocks: February, 2019 Report. 72 (Fort Collins, CO, April 2019).

[11] Agriculture, U. S. D. o. Code of Federal Regulations, Title 9, Part 56, Control of H5/H7 low pathogenic avian influenza. *Federal Register*, 206–215 (2019).

[12] Bonney PJ (2018). Evaluating the effect of the within-flock disease transmission rate on pre-movement active surveillance in low pathogenicity avian influenza infected flocks. Avian Dis..

[13] Becker NG (1989). Analysis of Infectious Disease Data.

[14] Ssematimba A (2018). Estimating the between-farm transmission rates for highly pathogenic avian influenza subtype H5N1 epidemics in Bangladesh between 2007 and 2013. Transbound. Emerg. Dis..

[15] Pillai S, Pantin-Jackwood M, Suarez D, Saif Y, Lee C-W (2010). Pathobiological characterization of low-pathogenicity H5 avian influenza viruses of diverse origins in chickens, ducks and turkeys. Adv. Virol..

[16] Iqbal M (2012). Selection of variant viruses during replication and transmission of H7N1 viruses in chickens and turkeys. Virology.

[17] Pantin-Jackwood MJ, Stephens CB, Bertran K, Swayne DE, Spackman E (2017). The pathogenesis of H7N8 low and highly pathogenic avian influenza viruses from the United States 2016 outbreak in chickens, turkeys and mallards. PLoS ONE.

[18] Saenz RA (2012). Quantifying transmission of highly pathogenic and low pathogenicity H7N1 avian influenza in turkeys. PLoS ONE.

[19] Spackman E (2010). The pathogenesis of low pathogenicity H7 avian influenza viruses in chickens, ducks and turkeys. Virol. J..

[20] Dundon WG, Maniero S, Toffan A, Capua I, Cattoli G (2007). Appearance of serum antibodies against the avian influenza nonstructural 1 protein in experimentally infected chickens and turkeys. Avian Dis..

[21] Morales AC (2008). Pathogenesis, Virus Shedding and Serologic Response in Selected Domestic Avian Species Against Low Pathogenic Avian Influenza (LPAI) Wild Bird Isolates.

[22] Homme, P., Easterday, B. & Anderson, D. Avian influenza virus infections. II. Experimental epizootiology of influenza A/Turkey/Wisconsin/1966 virus in turkeys. *Avian Dis.* 240–247 (1970). 5427232

[23] Preskenis L (2010). Characterization of Recent North American Low-Pathogencity Avian Influenza H7 Isolates in SPF Leghorns, Turkeys and Pekin Ducks.

[24] Spackman E (2002). Development of a real-time reverse transcriptase PCR assay for type A influenza virus and the avian H5 and H7 hemagglutinin subtypes. J. Clin. Microbiol..

[25] Comin A, Stegeman A, Marangon S, Klinkenberg D (2012). Evaluating surveillance strategies for the early detection of low pathogenicity avian influenza infections. PLoS ONE.

[26] Ssematimba, A. *et al.* Estimating within-flock transmission rate parameter for H5N2 highly pathogenic avian influenza virus in Minnesota turkey flocks during the 2015 epizootic. *Epidemiol. Infect.* **147** (2019). 10.1017/S0950268819000633 PMC6518789 31063119

[27] Bos ME, Nielen M, Koch G, Stegeman A, De Jong MC (2008). Effect of H7N1 vaccination on highly pathogenic avian influenza H7N7 virus transmission in turkeys. Vaccine.

[28] Bos ME (2010). Within-flock transmission of H7N1 highly pathogenic avian influenza virus in turkeys during the Italian epidemic in 1999–2000. Prevent. Vet. Med..

[29] Bos ME (2009). Back-calculation method shows that within-flock transmission of highly pathogenic avian influenza (H7N7) virus in the Netherlands is not influenced by housing risk factors. Prevent. Vet. Med..

[30] R: A language and environment for statistical computing (R Foundation for Statistical Computing, Vienna, Austria, 2020).

[31] Kernighan, B. W. & Ritchie, D. M. *The C Programming Language*. Vol. 2 (Prentice-Hall Englewood Cliffs, NJ, 1988).

[32] Wickham H (2016). ggplot2: Elegant Graphics for Data Analysis.

[33] Todd Weaver J (2016). A simulation-based evaluation of premovement active surveillance protocol options for the managed movement of turkeys to slaughter during an outbreak of highly pathogenic avian influenza in the United States. Avian Dis..

[34] Cardona, C. *et al.* An assessment of the risk associated with movement of broilers to market into, within, and out of a control area during a highly pathogenic avian influenza outbreak in the United States. 221 (Fort Collins, CO, October 2018).

[35] Raue A, Kreutz C, Theis FJ, Timmer J (2013). Joining forces of Bayesian and frequentist methodology: a study for inference in the presence of non-identifiability. Philos. Trans. R. Soc. A Math. Phys. Eng. Sci..

[36] Marjoram P, Molitor J, Plagnol V, Tavaré S (2003). Markov chain Monte Carlo without likelihoods. Proc. Natl. Acad. Sci..

[37] USDA-APHIS. Epidemiologic and other analyses of avian influenza affected poultry flocks: May 25, 2018 report. 36 (Fort Collins, CO, May 2018).

