# Supplementary material for: Estimating epidemiological parameters using diagnostic testing data from low pathogenicity avian influenza infected turkey houses
Source: Sci Rep. 2021 Jan 15;11:1602. doi: 10.1038/s41598-021-81254-z (PMC7810853; doi:10.1038/s41598-021-81254-z)
Supplement: Supplementary file 1 — Supplementary Information. [file 41598_2021_81254_MOESM1_ESM.docx]

Estimating epidemiological parameters using diagnostic testing data from low pathogenicity avian influenza infected turkey houses

Peter J. Bonney, Sasidhar Malladi, Amos Ssematimba, Erica Spackman, Mia Kim Torchetti, Marie Culhane, and Carol J. Cardona

**Supplementary Materials**

# Kandiyohi 2 diagnostic testing data

The diagnostic testing data for all five barns on Kandiyohi 2 used to estimate the contact rate and time of virus introduction is provided in Supplementary Table S1. Test results are provided from October 20^th^, 2019 until the flocks were sent to processing as part of controlled marketing following December 3^rd^, 2019. All houses first tested positive by rRT-PCR on November 5^th^. The last positive rRT-PCR result was observed on November 26^th^ in House 4.

Supplementary Table S1. Diagnostic testing data for all houses on Kandiyohi 2 consisting of the house size, sampling date, type of test, the composition of the sample, and test result.

| **Kandiyohi 2**  **House 1 – 9770 turkeys** | | | |
| --- | --- | --- | --- |
| Date | Test type | Sample | Result |
| 10/20/2018 | rRT-PCR | 1 pool of 11 swabs | 0/1 positive |
| 10/25/2018 | AGID | 9 serum samples | 0/9 positive |
| 11/01/2018 | rRT-PCR | 3 pools of 11 swabs each | 2/3 positive |
| 11/05/2018 | rRT-PCR | 1 pools of 11 swabs | 1/1 positive |
| 11/08/2018 | ELISA | 10 serum samples | 10/10 positive |
| 11/12/2018 | rRT-PCR | 1 pools of 11 swabs | 0/1 positive |
| 11/19/2018 | rRT-PCR | 3 pools of 11 swabs each | 0/3 positive |
| 11/26/2018 | rRT-PCR | 1 pools of 11 swabs | 0/1 positive |
| 12/03/2018 | rRT-PCR | 3 pools of 11 swabs each | 0/3 positive |
| **House 2 – 7260 turkeys** | | | |
| 10/20/2018 | rRT-PCR | 1 pools of 11 swabs | 0/1 positive |
| 10/25/2018 | AGID | 10 serum samples | 0/10 positive |
| 11/01/2018 | rRT-PCR | 3 pools of 11 swabs each | 3/3 positive |
| 11/05/2018 | rRT-PCR | 1 pools of 11 swabs | 1/1 positive |
| 11/08/2018 | ELISA | 10 serum samples | 1/10 positive |
| 11/12/2018 | rRT-PCR | 1 pools of 11 swabs | 1/1 positive |
| 11/16/2018 | ELISA | 10 serum samples | 10/10 positive |
| 11/19/2018 | rRT-PCR | 1 pools of 11 swabs | 0/1 positive |
| 11/26/2018 | rRT-PCR | 3 pools of 11 swabs each | 0/3 positive |
| 12/03/2018 | rRT-PCR | 1 pools of 11 swabs | 0/1 positive |
| **House 3 – 7360 turkeys** | | | |
| 10/20/2018 | rRT-PCR | 1 pools of 11 swabs | 0/1 positive |
| 10/25/2018 | AGID | 9 serum samples | 0/9 positive |
| 11/01/2018 | rRT-PCR | 3 pools of 11 swabs each | 3/3 positive |
| 11/05/2018 | rRT-PCR | 1 pools of 11 swabs | 1/1 positive |
| 11/08/2018 | ELISA | 10 serum samples | 0/10 positive |
| 11/12/2018 | rRT-PCR | 1 pools of 11 swabs | 1/1 positive |
| 11/15/2018 | ELISA | 10 serum samples | 10/10 positive |
| 11/19/2018 | rRT-PCR | 3 pools of 11 swabs each | 1/3 positive |
| 11/26/2018 | rRT-PCR | 1 pools of 11 swabs | 0/1 positive |
| 12/03/2018 | rRT-PCR | 3 pools of 11 swabs each | 0/3 positive |
| **House 4 – 7230 turkeys** | | | |
| 10/20/2018 | rRT-PCR | 1 pools of 11 swabs | 0/1 positive |
| 10/25/2018 | AGID | 9 serum samples | 0/9 positive |
| 11/01/2018 | rRT-PCR | 3 pools of 11 swabs each | 1/3 positive |
| 11/05/2018 | rRT-PCR | 1 pools of 11 swabs | 0/1 positive |
| 11/08/2018 | ELISA | 10 serum samples | 1/10 positive |
| 11/12/2018 | rRT-PCR | 1 pools of 11 swabs | 1/1 positive |
| 11/15/2018 | ELISA | 10 serum samples | 0/10 positive |
| 11/19/2018 | rRT-PCR | 3 pools of 11 swabs each | 3/3 positive |
| 11/26/2018 | rRT-PCR | 1 pools of 11 swabs | 1/1 positive |
| 11/29/2018 | ELISA | 10 serum samples | 10/10 positive |
| 12/03/2018 | rRT-PCR | 3 pools of 11 swabs each | 0/3 positive |
| **House 5 – 7300 turkeys** | | | |
| 10/20/2018 | rRT-PCR | 1 pools of 11 swabs | 0/1 positive |
| 10/25/2018 | AGID | 10 serum samples | 0/10 positive |
| 11/01/2018 | rRT-PCR | 3 pools of 11 swabs each | 3/3 positive |
| 11/05/2018 | rRT-PCR | 1 pools of 11 swabs | 1/1 positive |
| 11/08/2018 | ELISA | 10 serum samples | 10/10 positive |
| 11/12/2018 | rRT-PCR | 1 pools of 11 swabs | 0/1 positive |
| 11/19/2018 | rRT-PCR | 3 pools of 11 swabs each | 0/3 positive |
| 11/26/2018 | rRT-PCR | 1 pools of 11 swabs | 0/1 positive |
| 12/03/2018 | rRT-PCR | 3 pools of 11 swabs each | 0/3 positive |

# Summary of notation

Supplementary Table S2 provides a summary of the notation used in the estimation method for the time of virus introduction and adequate contact rate for the single house estimation approach. Similarly, Supplementary Table S3 provides a summary of the notation used in the multiple house estimation approach.

Supplementary Table S2. Variables included in the single house estimation approach for the adequate contact rate and time of virus introduction along with their descriptions.

| Variable | Description |
| --- | --- |
| *i* | Iterand for the transmission model simulation runs |
| *j* | Iterand for the rRT-PCR test events |
| $N_{pcr,j}^{swabs}$ | Number of swabs per pool in rRT-PCR test event *j* |
| $\rho_{I,ij}\left( \beta, t_{intro} \right)$ | Infection prevalence at the time of sampling for rRT-PCR test event *j* estimated from the *i*th transmission model simulation run given an adequate contact rate $\beta$ and time of virus introduction $t_{intro}$ |
| $p_{pcr,ij}$ | Probability of including at least one swab from an infectious bird in a single pooled sample taken as part of rRT-PCR test event *j* based on output from the *i*th transmission model simulation run |
| $N_{pcr,j}^{tests}$ | Total number of pooled rRT-PCR samples tested as part of test event *j* |
| $N_{pcr,j}^{pos}$ | Number of pooled rRT-PCR samples in test event *j* that tested positive |
| ${Se}_{pcr}$ | rRT-PCR test sensitivity |
| $P_{i}\left( N_{pcr,j}^{pos}\vert\beta, t_{intro} \right)$ | Probability of observing $N_{pcr,j}^{pos}$ positives given contact rate $\beta$, time of virus introduction$t_{intro}$, and output from the *i*th transmission model simulation run |
| *k* | Iterand for the serology test events |
| $N_{sero,k}^{tests}$ | Number of individual serum samples taken as part of test event *k* |
| $N_{sero,k}^{pos}$ | Number of positive serology samples in test event *k* |
| $\rho_{S,ik}\left( \beta, t_{intro} \right)$ | Seroprevalence at the time of sampling for serology test event *k* estimated from the *i*th transmission model simulation run given an adequate contact rate $\beta$ and time of virus introduction $t_{intro}$ |
| ${Se}_{sero}$ | AGID or ELISA test sensitivity |
| $P_{i}\left( N_{sero,k}^{pos} \vert\beta, t_{intro} \right)$ | Probability of observing $N_{sero,k}^{pos}$ positives given contact rate $\beta$, time of virus introduction$t_{intro}$, and output from the *i*th transmission model simulation run |
| $N_{iterations}$ | Number of transmission model simulation iterations performed |
| $N_{pcr}$ | Number of rRT-PCR test events |
| $N_{sero}$ | Number of serology test events |
| $l\left( x \vert\beta, t_{intro} \right)$ | The likelihood of observing the diagnostic test results given adequate contact rate $\beta$ and time of virus introduction $t_{intro}$ |
| $\pi(\beta, t_{intro})$ | The prior distribution for adequate contact rate $\beta$ and time of virus introduction $t_{intro}$ |
| $p\left( \beta, t_{intro}\vert x \right)$ | Posterior distribution of the contact rate and time of virus introduction |

Supplementary Table S3. Variables included in the multiple house estimation approach for the adequate contact rate and time of virus introduction along with their descriptions.

| Variable | Description |
| --- | --- |
| $l_{h}\left( x\vert\beta, t_{intro} \right)$ | The likelihood of observing the diagnostic test results from house *h* given adequate contact rate $\beta$ and time of virus introduction $t_{intro}$ |
| $t_{1}$ | The earliest time of virus introduction evaluated for a given house |
| $t_{max}$ | The latest time of virus introduction evaluated for a given house |
| $l_{h}\left( x\vert\beta\right)$ | The marginal contact rate likelihood for house *h* |
| $l_{H}\left( x\vert\beta\right)$ | The multiple house contact rate likelihood derived by multiplying the marginal contact rate likelihoods of the houses in set *H* |
| $l_{h_{1}}^{H}\left( x\vert\beta, t_{intro} \right)$ | The likelihood of observing the diagnostic test results from house $h_{1}$ given adequate contact rate $\beta$ and time of virus introduction $t_{intro}$ conditioned on the marginal contact rate likelihoods from the houses in set *H* |
| $p_{h_{1}}^{H}\left( \beta, t_{intro}\vert x \right)$ | The multiple house posterior distribution for house $h_{1}$ considering the marginal contact rate likelihoods of the houses in the set *H* |

# Validation results

The validation results are provided here for the three scenarios not included in the main text. The results from the simulated Kandiyohi 2 test data given a contact rate of 4.5 are provided in Supplementary Table S2. Similarly, the results from the simulated test data under the intensive testing scenario and a given contact rate of 1.0 are provided in Supplementary Table S3, and the results from the simulated test data under the intensive testing scenario and given contact rate of 4.5 are provided in Supplementary Table S4. The given time of virus introduction and adequate contact rate were enclosed in the estimated 95% CI in all scenarios. In general, the estimates were more accurate and had less uncertainty in the intensive testing scenarios. In the high contact rate scenarios, the estimates for the time of virus introduction were generally better than the comparable estimates in the low contact rate scenarios. The contact rate estimates, on the other hand, had estimated modes and upper 95% upper CI bounds entirely dependent on the upper bound of the prior distribution in the high contact rate scenarios. Last of all, the results suggest that the multiple house estimation approach can improve accuracy and reduce uncertainty in the parameter estimates.

Supplementary Table S4. Validation results given barns were infected 19, 6, and 3 days prior to and 1, 2, and 9 days after the date of the first test, assumed to have been performed on October 20. The time of virus introduction and adequate contact rate were estimated from simulated test data based on the Kandiyohi 2 test data and a contact rate of 4.5 contacts per day. Results are given for the single barn approach and multiple house approach.

| Given time of virus introduction and adequate contact rate | Single barn estimates mode (median; 95% CI) | Average mode (median; 95% CI) estimated with the contact rate distribution from 1 additional house | Mode (median; 95% CI) estimated with the contact rate distribution from 5 additional barns |
| --- | --- | --- | --- |
| Oct 1;  4.5 | Sep 27 (Sep 26;  Sep 16 – Oct 4);  5.6 (3.5; 0.9 – 5.8) | Sep 27 (Sep 26;  Sep 17 – Oct 4);  5.1 (3.6; 1.1 – 5.8) | Sep 28 (Sep 27;  Sep 19 – Oct 4);  6.0 (4.3; 1.7 – 5.9) |
| Oct 14;  4.5 | Oct 13 (Oct 11;  Sep 24 – Oct 15);  0.8 (3.00; 0.6 – 5.8) | Oct 13 (Oct 12;  Oct 3 – Oct 15);  3.2 (3.5; 1.0 – 5.8) | Oct 13 (Oct 13;  Oct 8 – Oct 15);  6.0 (4.2; 1.7 – 5.9) |
| Oct 17;  4.5 | Oct 16 (Oct 16;  Oct 8 – Oct 19);  5.9 (3.6; 0.9 – 5.8) | Oct 16 (Oct 16;  Oct 9 – Oct 19);  5.4 (3.8; 1.2 – 5.8) | Oct 17 (Oct 16;  Oct 13 – Oct 19);  6.0 (4.3; 1.8 – 5.9) |
| Oct 21;  4.5 | Oct 19 (Oct 19;  Oct 11 – Oct 24);  6.0 (3.7; 1.0 – 5.8) | Oct 19 (Oct 19;  Oct 12 – Oct 24);  5.5 (3.8; 1.2 – 5.8) | Oct 19 (Oct 19;  Oct 15 – Oct 24);  6.0 (4.3; 1.8 – 5.9) |
| Oct 22;  4.5 | Oct 21 (Oct 20;  Oct 10 – Oct 25);  6.0 (3.5; 0.9 – 5.8) | Oct 21 (Oct 20;  Oct 12 – Oct 25);  5.2 (3.7; 1.1 – 5.8) | Oct 21 (Oct 21;  Oct 16 – Oct 25);  6.0 (4.3; 1.8 – 5.9) |
| Oct 29;  4.5 | Oct 28 (Oct 28;  Oct 23 – Oct 30);  6.0 (4.1; 1.4 – 5.9) | Oct 28 (Oct 28;  Oct 23 – Oct 30);  6.0 (4.1; 1.5 – 5.9) | Oct 29 (Oct 28;  Oct 25 – Oct 30);  6.0 (4.5; 2.1 – 5.9) |

Supplementary Table S5. Validation results given barns were infected 19, 6, and 3 days prior to and 1, 2, and 9 days after the date of the first test, assumed to have been performed on October 20. The time of virus introduction and adequate contact rate were estimated from simulated test data based on a testing protocol of 3 pooled samples of 11 swabs each tested by rRT-PCR and 10 serum samples tested by AGID or ELISA every three days, and a contact rate of 1.0 contacts per day. Results are given for the single barn approach and multiple house approach.

| Given time of virus introduction and adequate contact rate | Single barn estimates mode (median; 95% CI) | Average mode (median; 95% CI) estimated with the contact rate distribution from 1 additional house | Mode (median; 95% CI) estimated with the contact rate distribution from 5 additional barns |
| --- | --- | --- | --- |
| Oct 1;  1.0 | Oct 9 (Oct 8;  Sep 26 – Oct 11);  5.6 (3.4; 0.8 – 5.8) | Oct 4 (Oct 3;  Sep 25 – Oct 9);  1.1 (1.4; 0.8 – 3.6) | Oct 2 (Oct 1;  Sep 27 – Oct 5);  1.1 (1.1; 0.9 – 1.5) |
| Oct 14;  1.0 | Oct 15 (Oct 14;  Oct 7 – Oct 20);  1.0 (1.2; 0.7 – 2.7) | Oct 15 (Oct 14;  Oct 8 – Oct 18);  1.0 (1.1; 0.8 – 2.1) | Oct 14 (Oct 14;  Oct 10 – Oct 17);  1.0 (1.1; 0.9 – 1.4) |
| Oct 17;  1.0 | Oct 18 (Oct 18;  Oct 10 – Oct 23);  1.0 (1.2; 0.7 – 2.8) | Oct 18 (Oct 17;  Oct 12 – Oct 21);  1.0 (1.2; 0.8 – 2.1) | Oct 17 (Oct 17;  Oct 13 – Oct 20);  1.0 (1.1; 0.9 – 1.4) |
| Oct 21;  1.0 | Oct 26 (Oct 25;  Oct 18 – Oct 29);  1.3 (1.8; 0.9 – 5.0) | Oct 23 (Oct 23;  Oct 17 – Oct 27);  1.2 (1.4; 0.8 – 2.9) | Oct 22 (Oct 21;  Oct 17 – Oct 24);  1.1 (1.2; 0.9 – 1.5) |
| Oct 22;  1.0 | Oct 26 (Oct 25;  Oct 18 – Oct 29);  1.2 (1.7; 0.8 – 4.6) | Oct 24 (Oct 24;  Oct 18 – Oct 28);  1.2 (1.3; 0.8 – 2.8) | Oct 23 (Oct 22;  Oct 18 – Oct 25);  1.1 (1.2; 0.9 – 1.5) |
| Oct 29;  1.0 | Oct 30 (Oct 29;  Oct 21 – Nov 3);  0.9 (1.1; 0.7 – 2.5) | Oct 30 (Oct 29;  Oct 23 – Nov 2);  1.0 (1.1; 0.7 – 2.0) | Oct 29 (Oct 29;  Oct 25 – Nov 1);  1.0 (1.1; 0.9 – 1.4) |

Supplementary Table S6. Validation results given barns were infected 19, 6, and 3 days prior to and 1, 2, and 9 days after the date of the first test, assumed to have been performed on October 20. The time of virus introduction and adequate contact rate were estimated from simulated test data based on a testing protocol of 3 pooled samples of 11 swabs each tested by rRT-PCR and 10 serum samples tested by AGID or ELISA every three days, and a contact rate of 4.5 contacts per day. Results are given for the single barn approach and multiple house approach.

| Given time of virus introduction and adequate contact rate | Single barn estimates mode (median; 95% CI) | Average mode (median; 95% CI) estimated with the contact rate distribution from 1 additional house | Mode (median; 95% CI) estimated with the contact rate distribution from 5 additional barns |
| --- | --- | --- | --- |
| Oct 1;  4.5 | Sep 30 (Sep 29;  Sep 17 – Oct 3);  5.6 (3.4; 0.8 – 5.8) | Sep 30 (Sep 29;  Sep 24 – Oct 3);  4.9 (4.0; 1.5 – 5.8) | Sep 30 (Sep 30;  Sep 27 – Oct 3);  5.6 (4.6; 2.5 – 5.9) |
| Oct 14;  4.5 | Oct 13 (Oct 12;  Oct 5 – Oct 14);  6.0 (3.8; 1.2 – 5.8) | Oct 13 (Oct 12;  Oct 8 – Oct 14);  5.1 (4.1; 1.7 – 5.8) | Oct 13 (Oct 13;  Oct 10 – Oct 15);  5.7 (4.7; 2.6 – 5.9) |
| Oct 17;  4.5 | Oct 16 (Oct 16;  Oct 12 – Oct 18);  6.0 (4.3; 1.8 – 5.9) | Oct 16 (Oct 16;  Oct 13 – Oct 18);  5.7 (4.4; 2.0 – 5.9) | Oct 17 (Oct 16;  Oct 14 – Oct 18);  6.0 (4.8; 2.8 – 5.9) |
| Oct 21;  4.5 | Oct 21 (Oct 20;  Oct 15 – Oct 22);  3.2 (3.7; 1.4 – 5.8) | Oct 20 (Oct 20;  Oct 16 – Oct 22);  4.2 (4.0; 1.7 – 5.8) | Oct 21 (Oct 20;  Oct 18 – Oct 22);  4.9 (4.6; 2.5 – 5.9) |
| Oct 22;  4.5 | Oct 21 (Oct 20;  Oct 16 – Oct 22);  3.3 (3.8; 1.4 – 5.8) | Oct 21 (Oct 21;  Oct 17 – Oct 22);  4.2 (4.0; 1.7 – 5.8) | Oct 21 (Oct 21;  Oct 19 – Oct 22);  4.9 (4.6; 2.5 – 5.9) |
| Oct 29;  4.5 | Oct 28 (Oct 28;  Oct 24 – Oct 30);  6.0 (4.4; 1.9 – 5.9) | Oct 28 (Oct 28;  Oct 25 – Oct 30);  5.8 (4.4; 2.0 – 5.9) | Oct 28 (Oct 28;  Oct 26 – Oct 30);  6.0 (4.8; 2.8 – 5.9) |

# Sensitivity analysis for the serology test sensitivity

A sensitivity analysis was performed for the estimate of the AGID or ELISA test sensitivity used in the estimation of the date of virus introduction and adequate contact. The baseline estimate of 1.00 was reduced to 0.98 in the sensitivity analysis. A comparison of the results of the time of virus introduction estimated from the Kandiyohi 2 test data under the baseline and sensitivity analysis estimates for the serology test sensitivity is given in Supplementary Table S7. Similarly, Supplementary Table S8 gives a comparison of the estimates for the adequate contact rate. There is relatively little difference in the results of the sensitivity analysis as compared to the baseline results.

Supplementary Table S7. Comparison of the mode, median, and 95% CI for the date of virus introduction estimated from the Kandiyohi 2 diagnostic testing data considering an AGID or ELISA test sensitivity of 1.00 and 0.98 evaluated as part of a sensitivity analysis.

|  | Estimated date of virus introduction  mode (median; 95% CI) | |
| --- | --- | --- |
| House | Serology test sensitivity 1.00 (Baseline) | Serology test sensitivity 0.98 |
| House 1 | October 18  (Oct 17; Oct 10 – Oct 21) | October 18  (Oct 17; Oct 10 – Oct 21) |
| House 2 | October 23  (Oct 21; Oct 7 – Oct 27) | October 26  (Oct 23; Oct 12 – Oct 28) |
| House 3 | October 21  (Oct 20; Oct 6 – Oct 26) | October 24  (Oct 22; Oct 11 – Oct 28) |
| House 4 | October 22  (Oct 20; Oct 6 – Oct 28) | October 26  (Oct 24; Oct 13 – Oct 30) |
| House 5 | October 18  (Oct 17; Oct 11 – Oct 22) | October 18  (Oct 17; Oct 11 – Oct 22) |

Supplementary Table S8. Comparison of the mode, median, and 95% CI for the adequate contact rate estimated from the Kandiyohi 2 diagnostic testing data considering an AGID or ELISA test sensitivity of 1.00 and 0.98 evaluated as part of a sensitivity analysis.

|  | Estimated adequate contact rate  mode (median; 95% CI) | |
| --- | --- | --- |
| House | Serology test sensitivity 1.00 (Baseline) | Serology test sensitivity 0.98 |
| House 1 | 6.0 (3.9; 1.2 – 5.8) | 6.0 (3.9; 1.2 – 5.8) |
| House 2 | 0.8 (1.2; 0.5 – 5.0) | 0.8 (1.3; 0.5 – 5.2) |
| House 3 | 0.8 (1.0; 0.5 – 4.1) | 0.8 (1.1; 0.5 – 4.3) |
| House 4 | 0.5 (0.6; 0.4 – 1.0) | 0.5 (0.6; 0.4 – 0.9) |
| House 5 | 6.0 (3.9; 1.2 – 5.8) | 6.0 (3.9; 1.2 – 5.8) |

# Sensitivity analysis for the number of initially infected birds

A sensitivity analysis was performed for the number of turkeys initially infected with LPAIV in non-index barns. Under the baseline scenario, the initial number of infected birds was randomly selected from the set of integers from one to ten for each transmission model simulation iteration. Under the sensitivity scenario, the random selection was made from the set of integers from one to fifty. Therefore, the sensitivity scenario allows for the possibility of larger numbers of birds being initially exposed to virus in the non-index barns as compared to the assumption under the baseline scenario.

The estimated time of virus introduction is given in Supplementary Table S9 and estimated adequate contact rate is given in Supplementary Table S10 for both scenarios. Little difference was observed in the results for the adequate contact between the two scenarios. Conversely, the results for time of virus introduction suggest that increasing the number of initially infected birds in a barn increases the likelihood of virus exposure occurring close to the time of the initial positive rRT-PCR test result. This is likely due to higher numbers of initially infected birds reducing the amount of time until there is explosive exponential growth in virus transmission in the infected flock.

Most notably, the estimates for the lower bound of the 95% CI for the time of virus introduction were considerably closer to the first rRT-PCR positive result leading to narrower 95% CI bounds. Therefore, reducing the uncertainty in the appropriate range for the number of initially infected birds has the potential to reduce uncertainty in the estimates for the time of virus introduction. As such, this is an area that warrants further investigation. For the purposes of the current study, the range of one to ten was used for the number of initially infected birds in non-index barns due to the more conservative (wider) estimates for the 95% CI.

Supplementary Table S9. Comparison of the mode, median, and 95% CI for the date of virus introduction estimated from the Kandiyohi 2 diagnostic testing data considering two different scenarios for the number of initially infected birds for those barns exposed after the likely index barns.

|  | Estimated date of virus introduction  mode (median; 95% CI) | |
| --- | --- | --- |
| House | Number of initially infected birds: Random selection from {1, 2, 3, …, 10} (Baseline) | Number of initially infected birds: Random selection from {1, 2, 3, …, 50} |
| House 2 | October 23  (Oct 21; Oct 7 – Oct 27) | October 27  (Oct 25; Oct 16 – Oct 29) |
| House 3 | October 21  (Oct 20; Oct 6 – Oct 26) | October 26  (Oct 25; Oct 15 – Oct 29) |
| House 4 | October 22  (Oct 20; Oct 6 – Oct 28) | October 29  (Oct 27; Oct 17 – Oct 31) |

Supplementary Table S10. Comparison of the mode, median, and 95% CI for the adequate contact rate estimated from the Kandiyohi 2 diagnostic testing data considering two different scenarios for the number of initially infected birds for those barns exposed after the likely index barns.

|  | Estimated adequate contact rate  mode (median; 95% CI) | |
| --- | --- | --- |
| House | Number of initially infected birds: Random selection from {1, 2, 3, …, 10} (Baseline) | Number of initially infected birds: Random selection from {1, 2, 3, …, 50} |
| House 2 | 0.8 (1.2; 0.5 – 5.0) | 0.8 (1.3; 0.5 – 5.1) |
| House 3 | 0.8 (1.0; 0.5 – 4.1) | 0.7 (1.1; 0.5 – 4.3) |
| House 4 | 0.5 (0.6; 0.4 – 1.0) | 0.5 (0.6; 0.4 – 0.8) |
